# Integrative analysis of LAG3 immune signature and identification of a LAG3-related genes prognostic signature in kidney renal clear cell carcinoma

**DOI:** 10.18632/aging.205476

**Published:** 2024-01-25

**Authors:** Jie Li, Chungan Liu, Hui Su, Hao Dong, Zhiqian Wang, Yuqi Wang, Peng Zhao, Chaowei Zhang, Yi Zhao, Xuezhen Ma

**Affiliations:** 1 Department of Oncology, Affiliated Qingdao Central Hospital of Qingdao University, Qingdao Cancer Hospital, Qingdao 266042, China; 2 Department of Oncology, Liaocheng City People’s Hospital, Liaocheng 252004, China; 3 Institute for Translational Medicine, The Affiliated Hospital of Qingdao University, College of Medicine, Qingdao University, Qingdao 266003, China

**Keywords:** LAG3, immunotherapy, signature, biomarker, KIRC

## Abstract

Immune checkpoint blockade (ICB) therapy has resulted in improved overall survival in kidney renal clear cell carcinoma (KIRC), but most treated patients fail to show durable clinical responses. Lymphocyte activation gene-3 (LAG3) is a novel inhibitory immune checkpoint, but its expression pattern, prognostic value, and immunological role in KIRC remain unknown. In this study, we utilized TCGA_KIRC RNA-sequencing data to analyze the relationship between LAG3 expression and clinical features. The single-cell sequencing data and tissue immunofluorescence are employed to investigate the subcellular localization of LAG3 in KIRC. Kaplan-Meier plotter, TIMER, and TISIDB were used to assess the association between LAG3 expression and prognosis, as well as its correlation with immune-related components. We constructed the LAG3 interaction network by using STRING, GeneMANIA, BioGRID, and HitPredict databases. We found that LAG3 is upregulated and correlates with poor prognostic phenotype in KIRC. LAG3 is predominantly expressed on exhausted CD8+ T cells and shows strong co-expression with PDCD1 in KIRC. Moreover, our findings indicated that LAG3 not only inhibits T cell activation but also potentially regulates cell adhesion in KIRC. In conclusion, our study implies that LAG3 can serve as a potential prognostic biomarker for KIRC. Furthermore, blocking both LAG3 and PDCD1 may alleviate resistance to anti-PDCD1 therapy, providing novel insights for immunotherapy decision-making in KIRC patients.

## INTRODUCTION

Kidney renal clear cell carcinoma (KIRC), which represents the predominant histological subtype of renal cell carcinoma (RCC), accounts for approximately 70% of RCC cases [1]. Despite its high prevalence, KIRC poses significant challenges in terms of treatment selection, mainly due to inherent resistance to conventional chemoradiation approaches. Furthermore, while immune checkpoint blockade (ICB) targeting the PD-1/PD-L1 and CTLA-4 axis has shown promising clinical responses in a subset of KIRC patients, considerable proportion of patients do not benefit substantially from immunotherapy due to the lack of target gene expression [2–4]. Considering the substantial economic burden and associated toxicities associated with cancer therapies, there is a pressing need to explore alternative strategies for improved management of KIRC. Recently, considerable focus has been devoted to exploring a second category of inhibitory receptors, including LAG-3, TIM-3, and TIGIT [5–7]. Identifying these and other research targets as well as a comprehensive understanding of their functions are essential for optimizing the efficacy of cancer immunotherapy strategies. Among the various potential targets identified to date, LAG-3 stands out as a particularly promising immune checkpoint for immunotherapy.

The gene encoding lymphocyte activation gene-3 (LAG3), also referred to as cluster of differentiation 223 (CD223), is positioned in proximity to the CD4 gene on chromosome 12 and exhibits structural similarities to CD4. It is prominently expressed on activated CD4+ and CD8+ T cells [8]. Studies have shown that soluble LAG-3 (sLAG-3), which is formed by cleavage of the linking peptide between the D4 structural domain and transmembrane structural domains of LAG-3 by a membrane-penetrating metalloproteinase, may limit the effectiveness of T cell immunological responses [9, 10]. LAG3 expression has been reported to be upregulated in tumor-infiltrating lymphocytes (TILs) from a wide range of other cancers, such as hepatocellular carcinoma, gastric cancer, and hematologic malignancies [11]. The remarkable clinical outcomes observed in the RELATIVITY-047 trial (NCT03470922) have led to the approval of the world’s first LAG-3 immunotherapy for the management of unresectable or metastatic melanoma [12]. As a promising therapeutic target, an in-depth understanding of the biological function and mechanism of action of LAG3 in different cancers may provide ideas for the further application and optimization of LAG3-targeted immunotherapy. In this study, we systematically analyzed the expression pattern, immunological role, and prognostic value of LAG3, and identified prognostic features associated with LAG3 in KIRC.

## RESULTS

### LAG3 is upregulated in KIRC and correlates with a poor prognostic phenotype

**Figure 1:**
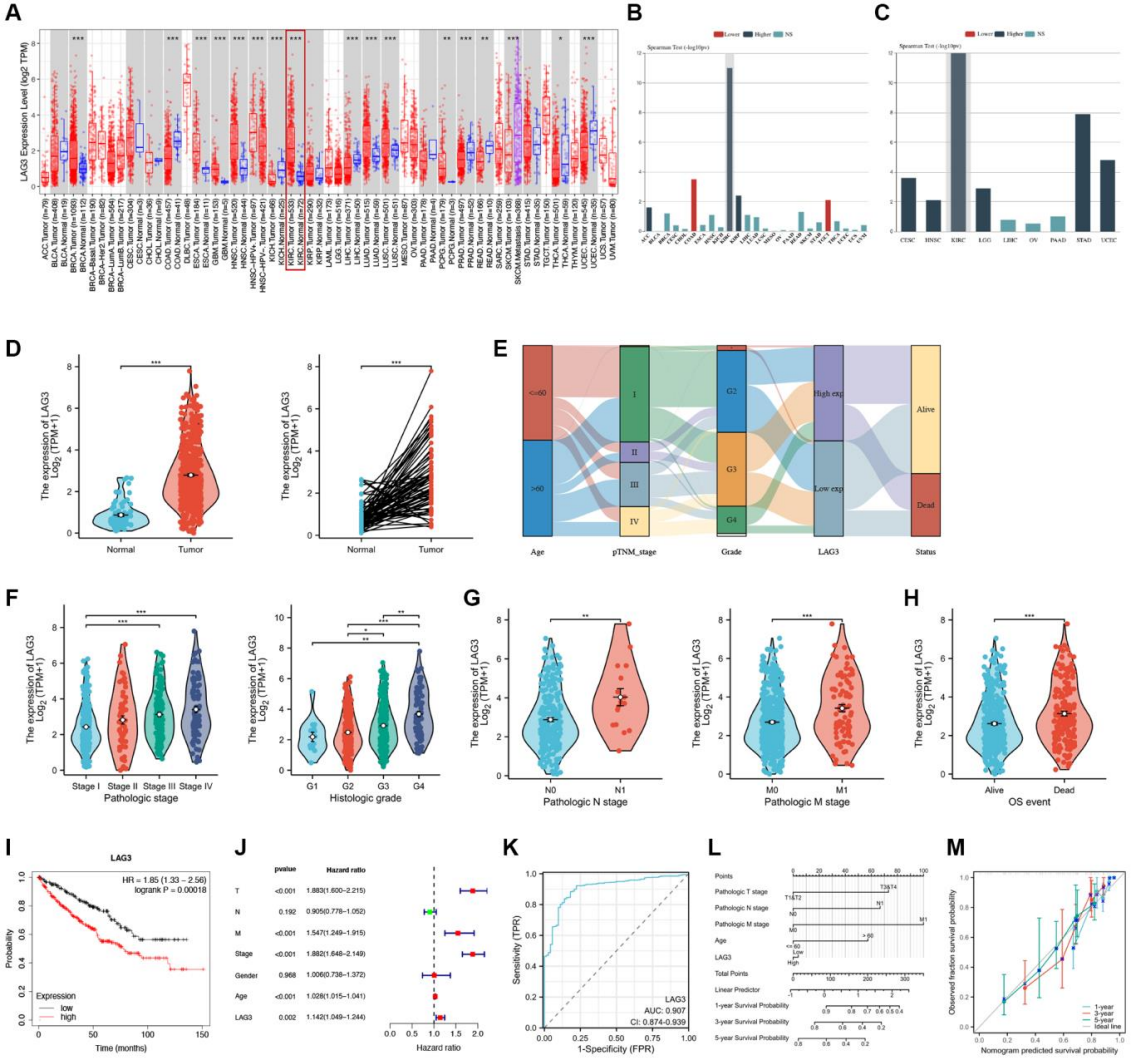
**The expression levels and prognostic values of LAG3 in KIRC.** (**A**) LAG3 mRNA expression level in diverse tumors and their adjacent normal tissues from TCGA. (**B**) Correlation between LAG3 expression and clinical stage in pan-cancer. (**C**) Correlation between LAG3 expression and clinical grade in pan-cancer. Blue bars represent higher level expression and are associated with later stage, and grade. (**D**) Comparison of LAG3 expression between KIRC cancer tissues and normal or matched normal tissues. (**E**) The trend of high and low expression of LAG3 for different clinical characteristics and survival status. LAG3 expression in different clinical and pathological features of KIRC, including pathologic stages, histologic grades (**F**), pathologic N stage, pathologic M stage (**G**), and OS event (**H**). (**I**) Kaplan–Meier curves of OS between high and low LAG3 expression groups. (**J**) Univariate Cox regression analysis of LAG3. (**K**) ROC curve to evaluate the diagnostic value of LAG3 expression in distinguishing KIRC tumor tissue from normal tissue. (**L**) Nomogram to predict the 1-year, 3-year, and 5-year OS of KIRC. (**M**) Nomogram calibration curve for the OS. Abbreviation: OS: overall survival. (^*^P < 0.05; ^**^P < 0.01; ^***^P < 0.001).

The TIMER database was used to analyze differential expression of LAG3 at pan-cancer level. Compared with normal tissues, LAG3 was highly overexpressed in certain cancer types including BRCA (Breast Invasive Carcinoma), ESCA (Esophageal Carcinoma), GBM (Glioblastoma), HNSC (Head and Neck Squamous Cell Carcinoma), LUAD (Lung Adenocarcinoma), LUSC (Lung Squamous Cell Carcinoma), PCPG (Pheochromocytoma and Paraganglioma), and KIRC, while down-regulation was observed in COAD (Colon Adenocarcinoma), KICH (Kidney Chromophobe), PRAD (Prostate Adenocarcinoma), LIHC (Liver Hepatocellular Carcinoma), READ (Rectum Adenocarcinoma), THCA (Thyroid Carcinoma), and UCEC (Uterine Corpus Endometrial Carcinoma) (Figure 1A). Although the assessment of LAG3 expression in KIRC was limited to 533 tumor samples and 72 adjacent normal tissue samples in the TIMER database, notable differences in LAG3 expression were observed between KIRC tumor tissues and normal tissues.

The TISIDB database was utilized to explore the association between LAG3 expression and clinical stage and grade of cancers. Notably, significant correlations between LAG3 expression and stage and grade were identified specifically in renal cancer (Figure 1B, 1C). Therefore, we focused on clarifying the functional role of LAG3 in KIRC. According to TCGA KIRC RNA-seq data, LAG3 was overexpressed in both paired and unpaired cancer tissues as compared to healthy controls (Figure 1D). To explore the clinical significance of LAG3 in KIRC, we initially investigated its expression pattern in relation to various clinical and pathological characteristics. We constructed Sankey plots to demonstrate trends in high and low LAG3 gene expression in KIRC patient samples with different clinical features and survival conditions (Figure 1E). The findings revealed elevated expression levels of LAG3 in advanced stages and higher grades KIRC (Figure 1F). Additionally, LAG3 overexpression was observed in lymph node metastasis and distant metastatic tissues when compared to normal tissues (Figure 1G). The same outcomes are demonstrated by detailed information regarding particular thresholds, namely that LAG3 high and low expression groups are statistically distinct in the T-stage, N-stage, M-stage, and AJCC-stage (Table 1).

**Table 1:** Association between LAG3 mRNA expression and clinicopathologic characteristics in KIRC.

**Total (*n* = 538)**		**Expression**		***P*-value**
		**LAG3 high (*n* = 132)**	**LAG3 low (*n* = 406)**	
**Gender**				
Male	352 (65.4%)	87 (65.9%)	265 (65.3%)	0.894
Female	186 (34.6%)	45 (34.1%)	141 (34.7%)	
**Age (years)**				
≥60	289 (53.7%)	75 (56.8%)	214 (52.7%)	0.776
<60	249 (46.3%)	57 (43.2%)	192 (47.3%)	
**T stage**				
T1	277 (51.5%)	44 (33.3%)	233 (57.4%)	<0.001
T2	71 (13.2%)	22 (16.7%)	49 (12.1%)	
T3	179 (33.3%)	60 (45.5%)	119 (29.3%)	
T4	11 (2.0%)	6 (4.5%)	5 (1.2%)	
**N stage**				
No	241 (44.8%)	67 (50.7%)	174 (42.8%)	0.04
N1	16 (3.0%)	8 (6.1%)	8 (2.0%)	
**M stage**				
M0	428 (79.5%)	95 (72.0%)	333 (82.1%)	0.037
M1	78 (14.5%)	34 (25.7%)	44 (10.8%)	
Unknown	32 (6.0%)	3 (2.3%)	29 (7.1%)	
**AJCC stage**				
I	271 (50.3%)	41 (31.1%)	230 (56.7%)	<0.001
II	59 (10.9%)	18 (13.6%)	41 (10.1%)	
III	123 (22.8%)	38 (28.8%)	85 (20.9%)	
IV	82 (15.2%)	34 (25.7%)	48 (11.8%)	
Unknown	3 (0.6%)	1 (0.8%)	2 (0.5%)	

Abbreviations: KIRC: kidney renal clear cell carcinoma; AJCC: American Joint Committee on Cancer.

High LAG3 expression was associated with more dead events (Figure 1H). Subsequent survival analysis to assess the prognostic value of LAG3 in KIRC revealed an association between high LAG3 and lower survival compared with that in the low expression group (*P* = 0.00018; Figure 1I). In contrast, there was no difference in the RFS between the high and low LAG3 expression groups (Supplementary Figure 1A). We then conducted univariate and multivariate Cox regression analysis to examine the influence of LAG3 on KIRC survival. In the univariate regression analysis, LAG3 expression, AJCC stage, tumor pathological stage (T), and the presence of metastasis (M) were all identified as significant prognostic risk factors for KIRC (Figure 1J). However, after all relevant factors were included in the multivariate regression analysis, LAG3 expression was not identified as an independent prognostic predictor of KIRC (hazard ratio (HR) = 1,054, *P* = 0.238; Supplementary Figure 1B).

Based on these discoveries, we applied receiver operating characteristic (ROC) curve analysis to evaluate the diagnostic accuracy of LAG3 expression in discriminating KIRC tumor tissue from normal tissue, as well as different clinicopathological characteristics. LAG3 exhibited remarkable discriminatory potential in distinguishing tumor tissue from normal tissue, as demonstrated by an impressive area under the curve (AUC) of 0.854 (95% confidence interval (CI): 0.874–0.939). These results highlight the potential of LAG3 as a promising diagnostic marker for KIRC (Figure 1K). In the comparisons of the G3&G4 versus (vs.) G&G2 groups, the T3&T4 vs. T1&T2 groups, the M1vs. M0 groups, and the N1 vs. N0 groups, the AUCs were 0.640 (95% CI: 0.592–0.689), 0.624 (95% CI: 0.577–0.671), 0.628 (95% CI: 0.558–0.697), and 0.697 (95% CI: 0.567–0.826), respectively (Supplementary Figure 1C–1F).

Taking into consideration LAG3 expression along with relevant clinicopathological characteristics, we employed the Cox regression algorithm to develop nomograms for predicting the 1-year, 3-year, and 5-year overall survival (OS) rates of KIRC patients. When compared to the ideal model, the calibration plots for the 1-, 3-, and 5-year OS rates demonstrated favorable predictive performance in the entire cohort. These results indicated the potential utility of the generated nomograms for accurate prediction of the survival outcomes of KIRC patients (Figure 1L, 1M). As an inhibitory immune checkpoint, high expression of LAG3 is associated with a positive response to immunotherapy due to its impact on the cytotoxic activity of immune cells against tumor cells. Moreover, elevated LAG3 expression may indicate a poorer response to conventional anticancer treatments, such as traditional chemotherapy. Our analysis of the role of LAG3 expression in predicting responses to common anticancer drugs supports this notion. The results demonstrate that most anti-tumor drugs, including sorafenib, exhibit higher drug sensitivity in the LAG3 low-expression group. This suggests that the expression level of LAG3 holds valuable reference significance in the selection of anti-tumor drugs (Supplementary Figure 2).

### LAG3 is predominantly expressed on exhausted CD8^+^ T cells and strongly co-expressed with PDCD1

**Figure 2:**
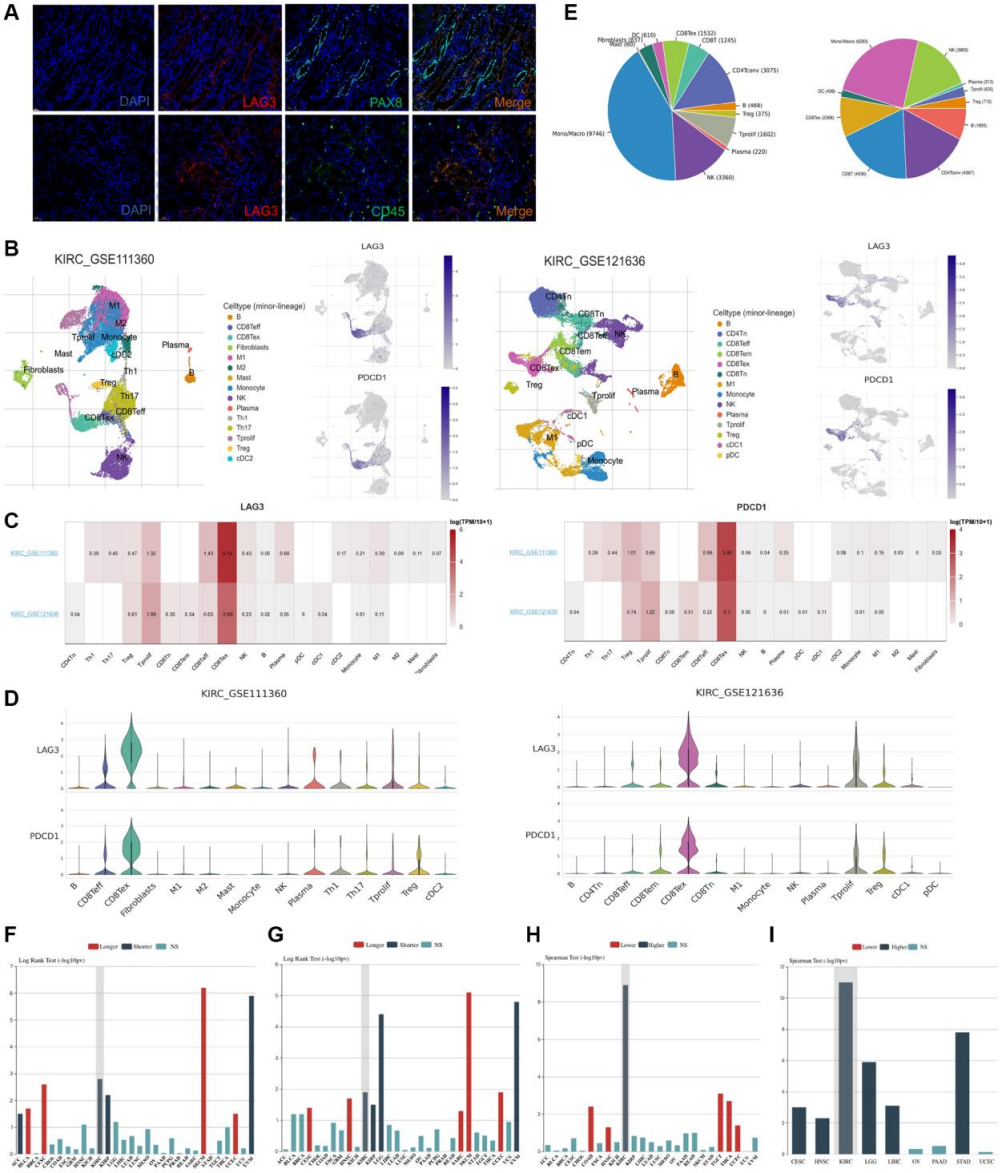
**LAG3 and PDCD1 cell type distribution in KIRC by single-cell seq datasets and correlation of their expression trends.** (**A**) Immunofluorescence staining of KIRC samples with DAPI (blue), LAG3 (red), CD45 (green), PAX8 (green), and the merged image (yellow) at 20× magnification, with a scale bar of 50 μm. (**B**) UMAP graph displays the distribution of cell types of LAG3 and PDCD1 expression in two single-cell seq datasets (KIRC_GSE111360 and KIRC_GSE121636). (**C**, **D**) LAG3 and PDCD1 expression levels across those two datasets in KIRC. (**E**) The pie chart shows the percentage of cell types in the two data sets. Bar graph showing the association between LAG3 (**F**) and PDCD1 (**G**) expression and OS, the correlation between PDCD1 expression and clinical stage (**H**) and grade (**I**) in multiple cancers.

After identifying the elevated expression of LAG3 in KIRC, we further analyzed its expression in subcellular types. Through immunofluorescence experiments, we observed that LAG3 expression is observed in immune cells (CD45+ cells) rather than in tumor cells (PAX8+ cells) (Figure 2A). To precisely identify the immune cell types expressing LAG3, we probed the cell-type localization of LAG3 expression in KIRC using two single-cell datasets. In both datasets, LAG3 was found to be expressed mainly on exhausted CD8^+^ T cells (CD8Tex cells) rather than on tumor cells or other types of immune cells compared and was also co-expressed with PDCD1(Figure 2B–2D). The pie charts show that CD8Tex occupies a non-negligible proportion of the KIRC immune cell composition (Figure 2E). Using the TISIDB database, we found that the expression patterns of LAG3 and PDCD1 showed similar association with OS, clinical stage, and grade in KIRC (Figure 2F–2I).

The early exhaustion of CD8^+^ T cells has been proposed as an indicator of a favorable clinical response to immune checkpoint inhibitor (ICI) therapy. Conversely, the emergence of late-stage exhaustion markers and post-treatment depletion markers has been associated with an unfavorable clinical outcome [13]. CD8Tex cells have the potential to restore their cytotoxic functionality, reverse T cell depletion, and enhance their capacity to eliminate tumors by inhibiting immunological checkpoints present on their cell surface [14]. From this, it can be inferred that LAG-3 and PDCD1 dual blockade may be a highly clinically active immunotherapy for KIRC.

### LAG3 expression is strongly positively associated with the majority of immune checkpoints and immune-related molecules

**Figure 3:**
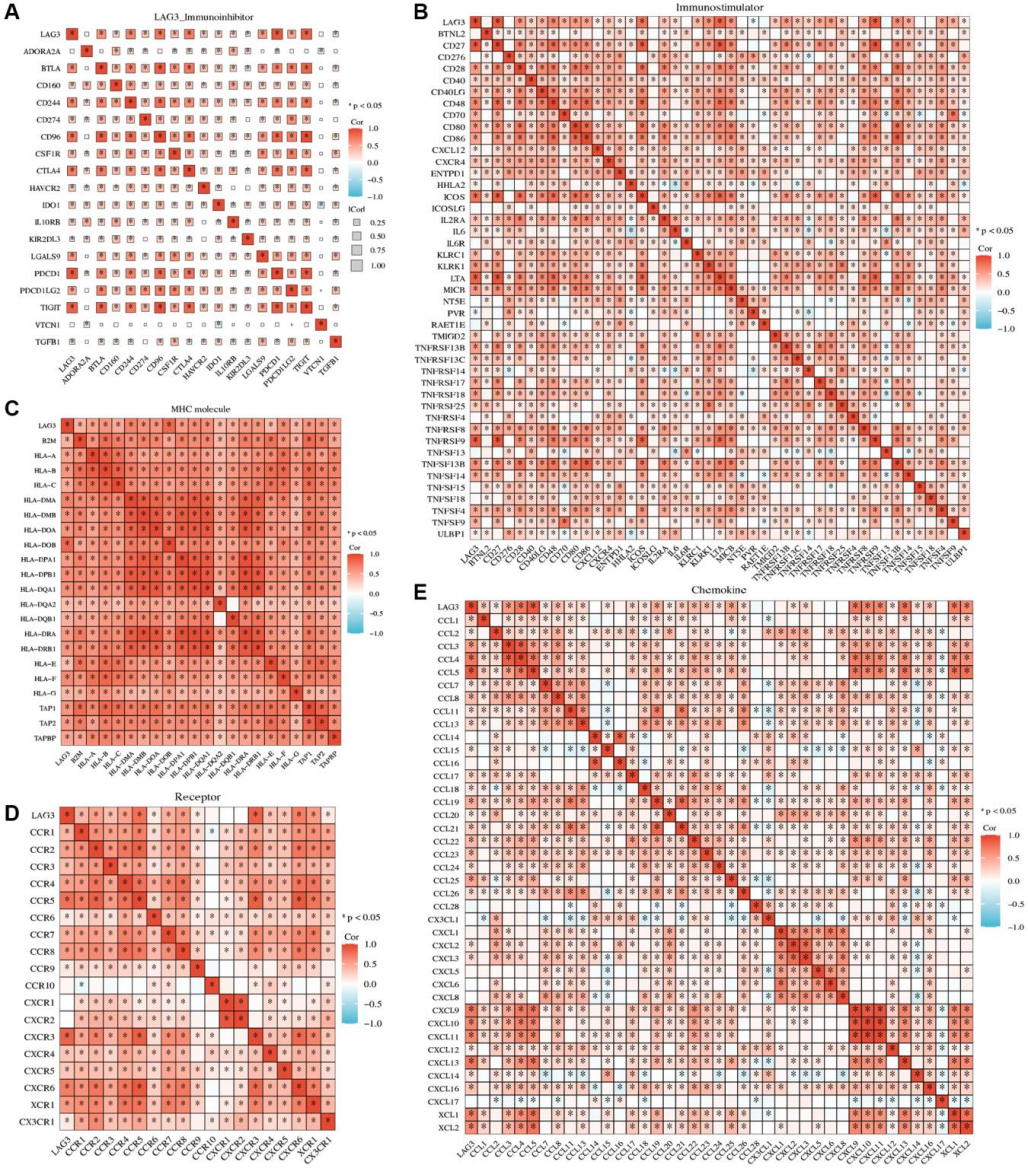
**Association between LAG3 expression and immune-related molecules.** The heatmap displays the correlation of LAG3 expression with immunoinhibitors (**A**), immunostimulators (**B**), MHC (**C**), receptors (**D**), and chemokines (**E**) in KIRC.

Based on the previous results, we further explored the correlation of LAG3 with other immune checkpoints and immune-related molecules in KIRC. Theoretically, immunoinhibitors tend to be highly expressed in the TME [15]. To gain a deeper understanding of the synergistic impact on immune response, we investigated potential associations between LAG3 and a diverse array of immune checkpoint inhibitors, encompassing PDCD1 (PD-1), CD274 (PD-L1), TIGIT, CTLA4, BTLA, and PDCD1LG2. The results indicated that LAG3 might modulate the antitumor immune response through co-regulation with these immune checkpoint molecules (Figure 3A). Furthermore, LAG3 expression was favorably linked with a wide variety of immunostimulators, including CD27, CD28, CD80, and CD86, indicating that LAG3 and immunostimulators interact to regulate immunological homeostasis (Figure 3B). LAG3 was also positively associated with almost all MHC-related genes, suggesting that LAG3 may interact with MHC molecules in addition to MHCII (Figure 3C). LAG3 exhibited positive associations with key receptors involved in the recruitment of effector tumor-infiltrating immune cells (TIICs), such as CVCR3-CVCR6, as well as chemokines including CCL3-CCL5 and CXCL9-CXCL13 (Figure 3D, 3E). Thus, further research into the connection between LAG3 and chemokines is warranted.

### Association between LAG3 expression and the cancer-immunity cycle

**Figure 4:**
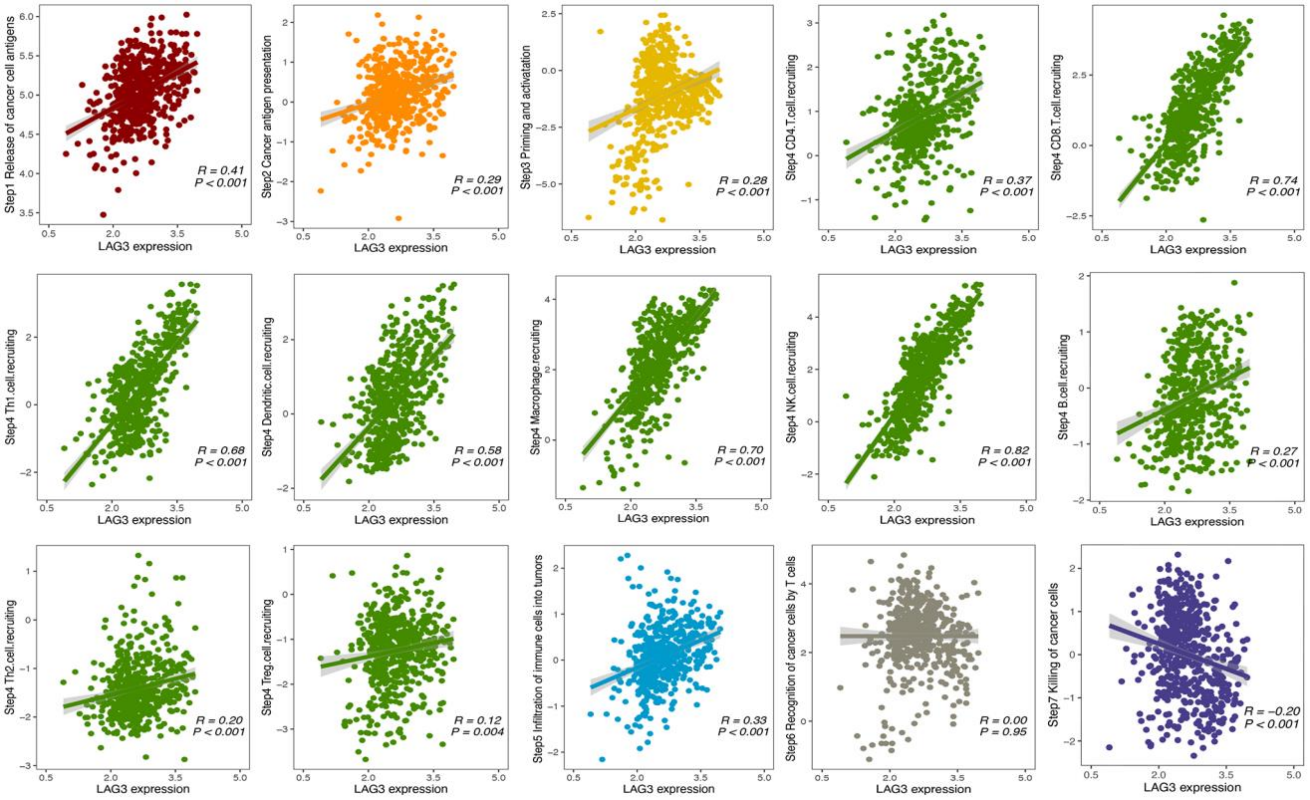
**Association between LAG3 expression and cancer immunity cycle (steps 1–7) in KIRC.** Association between LAG3 expression and cancer-immunity cycle (steps 1–7) in KIRC. Cancer-immunity cycle including release of cancer cell antigens (Step 1), cancer antigen presentation (Step 2), priming and activation (Step 3), trafficking of immune cells to tumors (Step 4), infiltration of immune cells into tumors (Step 5), recognition of cancer cells by T cells (Step 6) and killing of cancer cells (Step 7). The above 7-step immune activity scores were calculated for each sample of the KIRC dataset based on single-sample gene set enrichment analysis to assess the correlation between LAG3 expression and anti-cancer immune status.

Previous reports have shown that various chemokines and immunomodulators modulate the activity of the cancer-immunity cycle [16]. Therefore, we investigated a potential association of LAG3 with the cancer-immunity cycle in KIRC. We found that LAG3 expression was positively correlated with most immunity cycle activities, especially the trafficking of immune cells to tumors (step 4). LAG3 expression was strongly correlated with recruitment of CD8^+^ T cells (r = 0.74), NK cells (r = 0.82), macrophages (r = 0.70), and Th1 cells (r = 0.68), intermediately correlated with recruitment of dendritic cells (r = 0.58), and weakly correlated with recruitment of CD4^+^ T cells (r = 0.37), Th2 cells (r = 0.12), Treg cells (r = 0.12), B cells (r = 0.27). In accordance with our hypothesis, a negative correlation was observed between LAG3 expression and the efficacy of cancer cell eradication (step 7) (Figure 4), which confirmed its role as an inhibitory immune checkpoint that suppresses immune surveillance. Consistently, these results also revealed the role of LAG3 in promoting TIICs infiltration and shaping the inflammatory TME in KIRC.

### LAG3 expression is correlated with immune infiltration in KIRC

**Figure 5:**
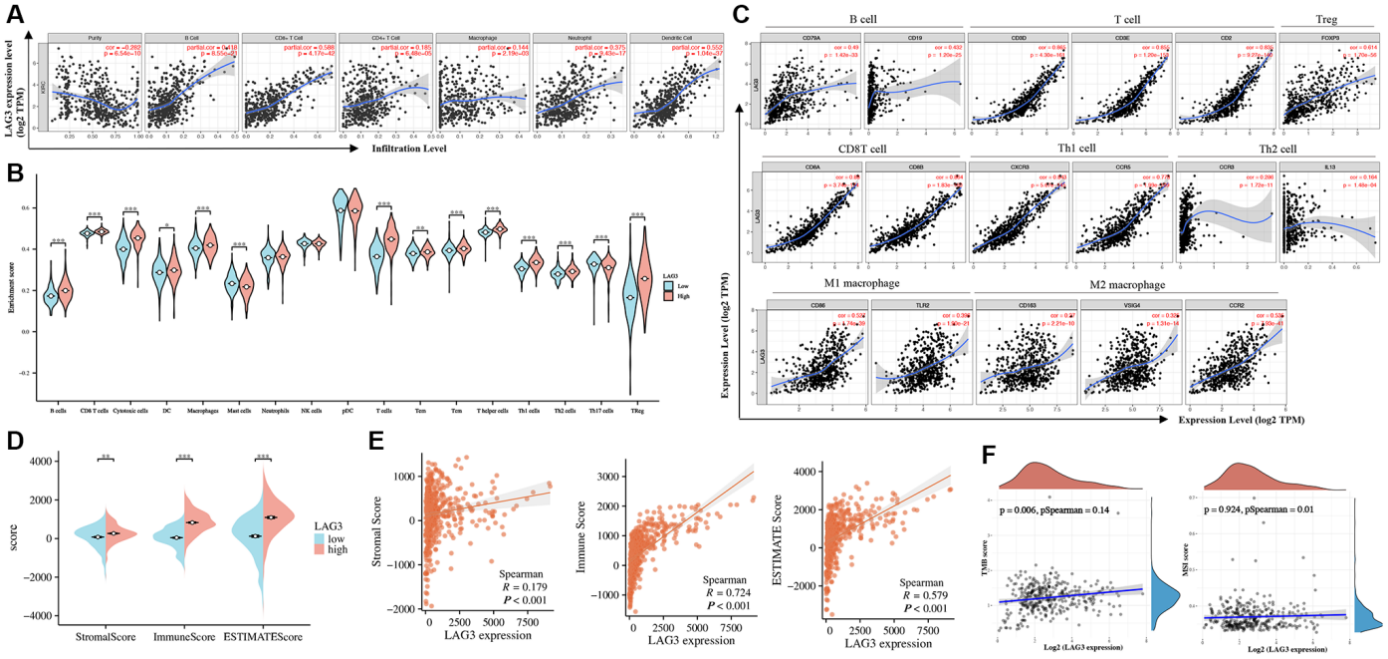
**Correlation between LAG3 expression and infiltration levels of immune cells in KIRC.** (**A**) Correlation analysis of LAG3 with the infiltration level of the six main immune cells after adjusting for purity. (**B**) The relationship between high and low expression of LAG3 and the level of immune cell infiltration. (**C**) The relation of LAG3 is analyzed with gene markers of immune cells. (**D**, **E**) Analysis of immune score, stromal score, and ESTIMATE score in high and low expression groups of LAG3. (**F**) Correlation analysis of LAG3 expression with TMB and MSI scores.

Analysis of the relationship between LAG3 and six main types of TIICs using TIMER revealed that LAG3 expression correlated positively correlates with CD8+ T cells, B cells, and dendritic cells (Figure 5A). Analysis of the abundance of infiltrating immune cells based on TCGA-KIRC data revealed that high LAG3 expression tended to imply richer infiltration by immune-activated TIICs, such as CD8+ T cells, CD4+ T cells, and Treg cells (Figure 5B). Additional exploration of LAG3 expression in immune cell markers demonstrated that LAG3 was significantly correlated with CD8+ T cells, Treg cells, and Th1 cells, and only moderately correlated with M1 macrophage cells, M2 macrophage cells, and Th2 cells (Figure 5C). Subsequent investigations revealed notable distinctions between the high- and low-LAG3 expression groups. Specifically, the high-LAG3 group exhibited elevated immune scores, stromal scores, and ESTIMATE scores when compared to the low-LAG3 expression group (Figure 5D). Correlation analysis further demonstrated a robust association between LAG3 expression and immune score (*p* < 0.001, r = 0.724), a moderate correlation with ESTIMATE score (*p* < 0.001, r = 0.579), and a weaker correlation with stromal score (*p* < 0.001, r = 0.179) (Figure 5E). In consideration of the significance of mutational burden in immunotherapy, we analyzed the correlation between LAG3 expression and Tumor Mutational Burden (TMB) as well as Microsatellite Instability (MSI). LAG3, as an immune checkpoint marker gene, is infrequently mutated. Similar to PD-1, MSI, and TMB, it serves as an independent factor for evaluating the efficacy of immunotherapy. Therefore, the expression level of LAG3 is not directly associated with MSI or TMB. Our results confirm this, as the correlation analysis indicates a weak association between LAG3 expression and TMB (*p* = 0.006, r = 0.14), and no statistically significant correlation with MSI scores (*p* = 0.924, r = 0.01) (Figure 5F).

### LAG3 interaction network construction and enrichment analysis

**Figure 6:**
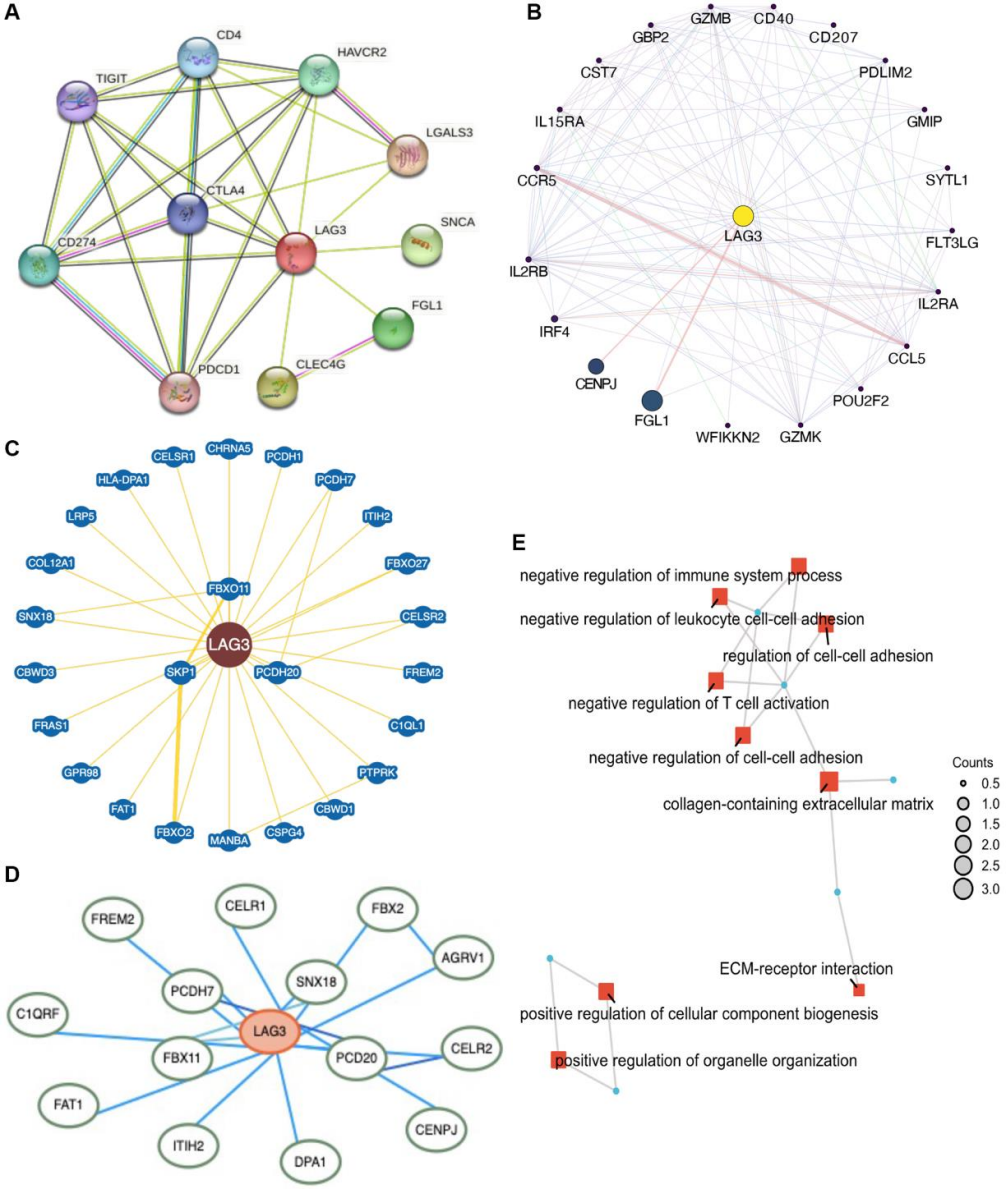
**Network construction and enrichment analysis for LAG3 co-expression genes in KIRC.** LAG3-related genes were analyzed via STRING (**A**), GeneMANIA (**B**), BioGRID (**C**), and HitPredict (**D**) sites. (**E**) GO and KEGG enrichment analysis.

Using STRING, GeneMANIA, BioGRID, and HitPredict to screen and collect LAG3 co-expressed genes, we identified eight associated genes in the intersection to better characterize the function of LAG3 in KIRC (Figure 6A–6D, Supplementary Table 1). The co-expressed genes associated with LAG3 were further examined by Gene Ontology (GO) and Kyoto Encyclopedia of Genes and Genomes (KEGG) enrichment analyses, providing valuable insights into the underlying biological mechanisms (Figure 6E). The results revealed significant enrichment in key biological processes such as T-cell activation and cell-cell adhesion, indicating that LAG3 cannot only blocks T cell activation but is also responsible for cell adhesion in KIRC.

### Construction of a prognostic model of LAG3-related genes

**Figure 7:**
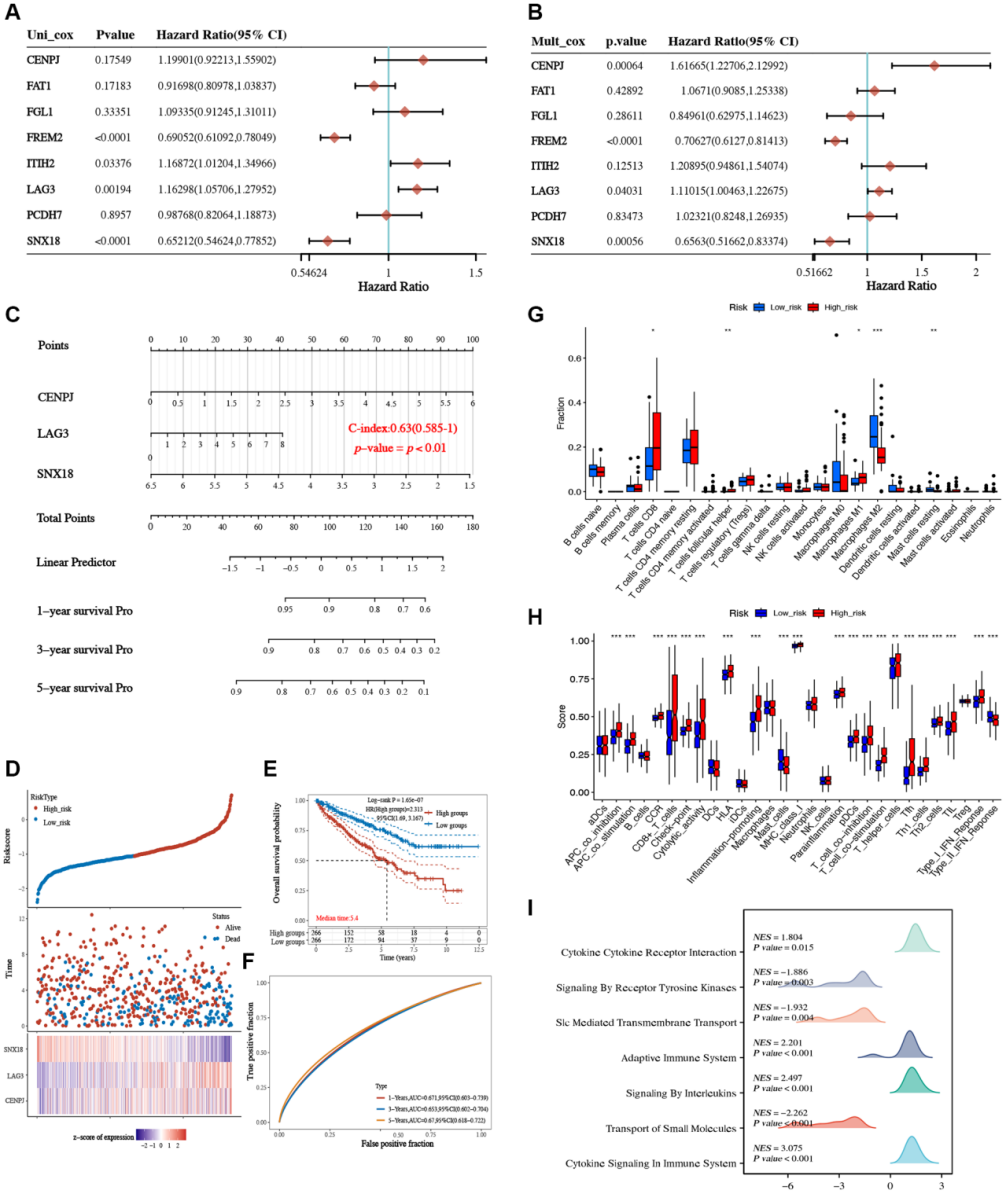
**Construction and validation of a prognostic model based on LAG3-related genes.** Univariate (**A**) and multivariate (**B**) Cox regression analysis of LAG3-related genes. (**C**) Nomogram to predict the 1-year, 3-year, and 5-year OS of KIRC. (**D**) Riskscore scatterplot, survival status scatterplot, and model gene expression heatmap. (**E**) Survival curve of high/low-risk score. (**F**) ROC curves of the prognostic model at 1-year, 3-years, and 5-years. (**G**) The box plot depicting the immune cell infiltration levels in the high- and low-risk groups. (**H**) Box plot of immune pathway scores in the high- and low-risk groups. (**I**) Mountain plots of GSEA for the high- and low-risk groups. (^*^P < 0.05; ^**^P < 0.01; ^***^P < 0.001).

Univariate and multivariate analyses of the eight LAG3-related genes revealed that CENPJ, LAG3, and SNX18 were independent prognostic variables for KIRC patients (Figure 7A, 7B). Based on this information, we developed a predictive nomogram that demonstrated favorable performance when compared to the ideal model in terms of the rates of survival at 1-, 3-, and 5-year survival rates (Figure 7C, Supplementary Figure 3A). A predictive signature consisting of these three prognostic biomarkers (CENPJ, LAG3, and SNX18) was created using LASSO Cox regression analysis based on these potential prognostic biomarkers (Supplementary Figure 3B, 3C). Following the calculation of the risk score for KIRC patients using the equation ((0.4692× CENPJ expression) + (0.1458 × LAG3 expression) + (−0.5678 × SNX18 expression)), the KIRC patient cohort was segregated into high-risk and low-risk groups. The data analysis revealed that KIRC patients with elevated risk scores exhibited an unfavorable prognosis (Figure 7D, 7E). Furthermore, the area under the receiver operating characteristic (ROC) curves at 1, 3, and 5 years were determined as 0.671, 0.653, and 0.67, respectively, thereby illustrating the robust predictive capacity of this feature in predicting outcomes for KIRC patients (Figure 7F). Through further analysis of immune features, we observed differences in the composition of immune cells between the high-risk and low-risk groups (Supplementary Figure 3D). In comparison to the low-risk group, the high-risk group demonstrated higher levels of immune cell infiltration and association with immune pathways (Figure 7G, 7H). The GSEA analysis also revealed enrichment in immune-related pathways for the high-risk group (Figure 7I). This indicates that there is a difference in the immune status between the high-risk and low-risk groups, suggesting that the high-risk group may have potential predictive value for the immunotherapeutic responsiveness of KIRC.

## DISCUSSION

KIRC exhibits significant heterogeneity in clinical manifestations and prognosis [17]. Currently, reliable biomarkers for treatment and prognosis are lacking, necessitating further research to identify novel and practical markers [18]. Although several studies have explored prognostic markers for KIRC, their clinical application remains limited [19–21]. Compared to other tumor types, KIRC demonstrates higher immune cell cytotoxicity and immune infiltration scores [22, 23]. However, the efficacy of immune checkpoint inhibitors targeting PD-1/PD-L1 and CTLA-4 in KIRC treatment is suboptimal, with low complete response rates for monotherapy [24]. Consequently, the development of novel immune checkpoints and the exploration of their combination or sequential use have emerged as crucial avenues for improving the effectiveness of immunotherapy in KIRC. Currently, immunotherapy targeting LAG3 has gained prominence as a hot topic in research [25]. However, the efficacy and feasibility of LAG3 antibody therapy in KIRC treatment require further evaluation. Therefore, we conducted a comprehensive investigation into the functional role of LAG3 in KIRC.

Our analysis showed that LAG3 was upregulated in tumor tissues, especially in late-staged, high-grade KIRC. In accordance with previous studies, we also found that elevated LAG3 expression was linked to an unfavorable prognosis and was identified as an independent prognostic indicator in KIRC [26]. Conversely, in gastric cancer, lymphoma, and colorectal cancer, high LAG3 expression has been associated with a favorable prognosis [27]. Consequently, it is crucial to investigate the role of LAG3 in distinct cancer types individually.

LAG3 was expressed on a variety of immune cell types. Analysis of KIRC single-cell sequencing data demonstrated that LAG3 is expressed mainly by CD8Tex cells in KIRC and highly co-expressed with PD-1. Previous studies showed that LAG3 expression is strongly correlated with PD-1 expression only in papillary RCC subtypes [28], indicating that blocking both LAG3 and PD-1 in RCC may alleviate the resistance to anti-PD-1 blockade.

Multiple potential therapies that target LAG3 are now being investigated in clinical studies of various human malignancies [29]. Additionally, seven bispecific antibodies that target LAG3 and PD-1 or CTLA-4 have recently been introduced to the clinic [29], although notable clinical efficacy has not yet been observed. Therefore, thorough investigation of the impacts of blocking LAG3 on the functions of CD8^+^T cells and other immune cell types, and its mechanism of synergistic interaction with PD-1 are crucial for the development of anti-LAG3 immunotherapy strategies.

Studies evaluating the predictive potential of LAG3 in cancer patients are an active area of research. The value of LAG3-related protein-protein interactions (PPIs) in KIRC prognosis has not been investigated in other studies. To this end, we first established an interaction network for LAG3 co-expression genes in KIRC and performed functional enrichment analysis. We discovered that LAG3 not only inhibits T cell activation, but might also regulate cell adhesion in KIRC, which has not yet been reported. Indeed, this requires further molecular experimental investigation.

Through constructing a risk model, we also identified that the co-expression of three genes with LAG3 holds significant value in predicting prognosis and immune therapy outcomes, albeit requiring further biological evidence for support. Overall, our study offers some evidence to support the use of LAG-3 as a biomarker for the evaluation of prognostic and treatment in KIRC.

## METHODS

### Data acquisition and expression analysis

The RNA-sequencing data of KIRC obtained from The Cancer Genome Atlas (TCGA) database were subjected to normalization, tumor purity assessment, and gene annotation conversion processes. These operations yielded expression data for 610 samples (Tumor: 538, Normal: 72). Corresponding clinical data for tumor samples were also obtained from the TCGA-KIRC dataset. All data were then processed with the “TCGAbiolinks” in the R package. All gene expression values underwent a logarithmic transformation and subsequent normalization process. The R package “ggalluvial” was used to construct the Sankey diagram. The R package “ggstatsplot” was employed to construct the correlation ridge plots. We examined the cell-type localization of LAG3 and PDCD1 expression in KIRC using single-cell sequencing datasets from Tumor Immune Single-cell Hub 2 (TISCH2) [30], an online site that provides detailed cell-type annotations.

### Drug sensitivity analysis

Using the R package “pRRophetic” algorithm, we calculated the half-maximal inhibitory concentration (IC50) of commonly used drugs for KIRC patients in the LAG3 high and low expression groups. The results were visualized using the R packages “ggplot2” and “ggpubr”.

### Immune feature analysis

The list of immunoinhibitors, immunostimulators, MHC molecules, chemokines, and receptor molecules was obtained from the TISIDB online website, a comprehensive repository portal for tumor immune system interactions [31]. Analysis of LAG3 correlation with tumor-infiltrating immune cells (TIICs) and cell surface molecular markers was done using the TIMER database, a website that comprehensively analyzes immune infiltration in various cancers [32]. Immune infiltration in the high and low LAG3 expression groups in TCGA-KIRC RNA-seq was compared using the “ssGSEA” algorithm provided in the R package “GSVA”. The ESTIMATE algorithm [33] was used to examine the immune score, stromal score, and tumor purity, which provides valuable information about the presence and composition of immune cells and stromal cells within the tumor microenvironment (TME). Furthermore, we investigated the association between LAG3 expression and the seven-step cancer immunity cycle, which represents the intricate interplay between immune responses and the fate of tumor cells, using the Tumor Immune Profiling (TIP) approach [34]. The obtained results were visualized using the “ggpubr” and “ggplot2” in the R packages.

### Protein-protein interaction network construction and correlation analysis

Genes co-expressed with LAG3 were obtained by searching the STRING, GeneMANIA, BioGRID, and HitPredict databases [35–37]. After screening the recurring genes, eight LAG3-related genes were identified. Gene ontology (GO) and Kyoto Encyclopedia of Genes and Genomes (KEGG) enrichment analyses were conducted to investigate the function of the LAG3-dominated gene interaction network using “clusterProfiler” package in R software.

### Prognostic analysis and establishment of a LAG3-related genes risk signature

We utilized the Kaplan-Meier plotter [38] to evaluate the association between differential expression of LAG3 and overall survival (OS) in KIRC patients. We also performed univariate and multivariate Cox regression analyses to assess the prognostic significance of LAG3. The results were visualized as forest plot generated using R package “survivor and survminer”. A nomogram was produced using the “rms” software and the total recurrence rate in 1-, 3-, and 5-year OS were forecasted based on the results of multivariate Cox proportional hazards analysis.

The regression coefficient for the three most statistically significant prognostic genes were obtained from a multivariate Cox proportional hazards regression model. To perform the LASSO Cox regression, we utilized the package “glmnet” in R and estimated the penalty parameter through 10-fold cross-validation. Subsequently, we calculated the risk score using the following formula:


Riskscore=∑Expi × βi

Based on the median risk score, the patients were categorized as high- and low-risk groups. We then conducted Kaplan-Meier survival analysis and employed a log-rank test to assess the statistical significance of survival outcomes between these two groups.

### Functional state analysis of LAG3-related gene risk features

Gene Set Enrichment Analysis (GSEA) analysis was performed using the R package “clusterProfiler” to characterize the potential biological functions of genes between the high-risk and low-risk groups. The reference gene set used was c2.cp.all.v2022.1.Hs.symbols.gmt (All Canonical Pathways). Visualization of enrichment analysis results was conducted using the “ggplot2” package. Single-sample Gene Set Enrichment Analysis (ssGSEA) was carried out with the R package “GSVA” to assess the infiltration levels of immune cell types and immune pathways between the high-risk and low-risk groups.

### Immunofluorescence (IF) staining

After obtaining the patient’s consent and approval from the Ethics Committee of Qingdao Central Hospital (KY202318401), we obtained pathological sections from KIRC patients. Anti-LAG3 antibody (ab209236, 1:100 dilution), anti-CD45 antibody (ab40763, 1:100 dilution), and anti-PAX8 antibody (ab191870, 1:1000 dilution) were purchased from Abcam (Cambridge, UK). BSA (G5001), PBS (G0002), EDTA (G1203), and DAPI (G1012) were purchased from Servicebio (Wuhan, China). Paraffin-embedded tissue sections underwent xylene deparaffinization, followed by ethanol dehydration. Microwave antigen retrieval (EDTA buffer, pH 9.0, 10 minutes) was performed, and non-specific binding was blocked with BSA for 30 minutes. Anti-CD45, anti-PAX8, and anti-LAG3 antibodies were applied and incubated overnight at 4°C. After three PBS washes, sections were exposed to secondary antibodies for 50 minutes at room temperature under light protection. DAPI staining (10 minutes) for cell nuclei and a 5-minute application of autofluorescence quenching reagent were followed. Sections were then mounted on slides for microscopy, and images were captured using a fluorescence microscope.

### Statistical analysis

Differences between groups of data were assessed using the Wilcoxon test or Kruskal–Wallis test as appropriate. Pearson correlation analysis was conducted to determine correlations between variables. Kaplan–Meier analysis and log-rank tests were conducted for survival analysis. Univariate and multivariate Cox regression analyses were employed to identify independent prognostic factors. All statistical analyses were performed using R software (version 4.0.4) and *P* < 0.05 was considered to indicate statistical significance.

## Supplementary Materials

Supplementary Figures

Supplementary Table 1

## References

[1] Díaz-Montero CM, Rini BI, Finke JH. The immunology of renal cell carcinoma. Nat Rev Nephrol. 2020; 16:721–35. 10.1038/s41581-020-0316-3 32733094

[2] Rijnders M, de Wit R, Boormans JL, Lolkema MPJ, van der Veldt AAM. Systematic Review of Immune Checkpoint Inhibition in Urological Cancers. Eur Urol. 2017; 72:411–23. 10.1016/j.eururo.2017.06.012 28645491

[3] Choueiri TK, Motzer RJ. Systemic Therapy for Metastatic Renal-Cell Carcinoma. N Engl J Med. 2017; 376:354–66. 10.1056/NEJMra1601333 28121507

[4] Galon J, Bruni D. Tumor Immunology and Tumor Evolution: Intertwined Histories. Immunity. 2020; 52:55–81. 10.1016/j.immuni.2019.12.018 31940273

[5] Andrews LP, Yano H, Vignali DAA. Inhibitory receptors and ligands beyond PD-1, PD-L1 and CTLA-4: breakthroughs or backups. Nat Immunol. 2019; 20:1425–34. 10.1038/s41590-019-0512-0 31611702

[6] Anderson AC, Joller N, Kuchroo VK. Lag-3, Tim-3, and TIGIT: Co-inhibitory Receptors with Specialized Functions in Immune Regulation. Immunity. 2016; 44:989–1004. 10.1016/j.immuni.2016.05.001 27192565 PMC4942846

[7] Qin S, Xu L, Yi M, Yu S, Wu K, Luo S. Novel immune checkpoint targets: moving beyond PD-1 and CTLA-4. Mol Cancer. 2019; 18:155. 10.1186/s12943-019-1091-2 31690319 PMC6833286

[8] Triebel F, Jitsukawa S, Baixeras E, Roman-Roman S, Genevee C, Viegas-Pequignot E, Hercend T. LAG-3, a novel lymphocyte activation gene closely related to CD4. J Exp Med. 1990; 171:1393–405. 10.1084/jem.171.5.1393 1692078 PMC2187904

[9] Li N, Workman CJ, Martin SM, Vignali DA. Biochemical analysis of the regulatory T cell protein lymphocyte activation gene-3 (LAG-3; CD223). J Immunol. 2004; 173:6806–12. 10.4049/jimmunol.173.11.6806 15557174

[10] Buisson S, Triebel F. LAG-3 (CD223) reduces macrophage and dendritic cell differentiation from monocyte precursors. Immunology. 2005; 114:369–74. 10.1111/j.1365-2567.2004.02087.x 15720438 PMC1782096

[11] Long L, Zhang X, Chen F, Pan Q, Phiphatwatchara P, Zeng Y, Chen H. The promising immune checkpoint LAG-3: from tumor microenvironment to cancer immunotherapy. Genes Cancer. 2018; 9:176–89. 10.18632/genesandcancer.180 30603054 PMC6305110

[12] Tawbi HA, Schadendorf D, Lipson EJ, Ascierto PA, Matamala L, Castillo Gutiérrez E, Rutkowski P, Gogas HJ, Lao CD, De Menezes JJ, Dalle S, Arance A, Grob JJ, et al, and RELATIVITY-047 Investigators. Relatlimab and Nivolumab versus Nivolumab in Untreated Advanced Melanoma. N Engl J Med. 2022; 386:24–34. 10.1056/NEJMoa2109970 34986285 PMC9844513

[13] Chow A, Perica K, Klebanoff CA, Wolchok JD. Clinical implications of T cell exhaustion for cancer immunotherapy. Nat Rev Clin Oncol. 2022; 19:775–90. 10.1038/s41571-022-00689-z 36216928 PMC10984554

[14] Dolina JS, Van Braeckel-Budimir N, Thomas GD, Salek-Ardakani S. CD8^+^ T Cell Exhaustion in Cancer. Front Immunol. 2021; 12:715234. 10.3389/fimmu.2021.715234 34354714 PMC8330547

[15] Gajewski TF, Corrales L, Williams J, Horton B, Sivan A, Spranger S. Cancer Immunotherapy Targets Based on Understanding the T Cell-Inflamed Versus Non-T Cell-Inflamed Tumor Microenvironment. Adv Exp Med Biol. 2017; 1036:19–31. 10.1007/978-3-319-67577-0_2 29275462 PMC6693322

[16] Chen DS, Mellman I. Oncology meets immunology: the cancer-immunity cycle. Immunity. 2013; 39:1–10. 10.1016/j.immuni.2013.07.012 23890059

[17] Rysz J, Franczyk B, Ławiński J, Gluba-Brzózka A. Characteristics of Clear Cell Papillary Renal Cell Carcinoma (ccpRCC). Int J Mol Sci. 2021; 23:151. 10.3390/ijms23010151 35008576 PMC8745490

[18] Lopez-Beltran A, Henriques V, Cimadamore A, Santoni M, Cheng L, Gevaert T, Blanca A, Massari F, Scarpelli M, Montironi R. The Identification of Immunological Biomarkers in Kidney Cancers. Front Oncol. 2018; 8:456. 10.3389/fonc.2018.00456 30450335 PMC6225533

[19] Brooks SA, Brannon AR, Parker JS, Fisher JC, Sen O, Kattan MW, Hakimi AA, Hsieh JJ, Choueiri TK, Tamboli P, Maranchie JK, Hinds P, Miller CR, et al. ClearCode34: A prognostic risk predictor for localized clear cell renal cell carcinoma. Eur Urol. 2014; 66:77–84. 10.1016/j.eururo.2014.02.035 24613583 PMC4058355

[20] Xu F, Xu L, Wang Q, An G, Feng G, Liu F. Clinicopathological and prognostic value of programmed death ligand-1 (PD-L1) in renal cell carcinoma: a meta-analysis. Int J Clin Exp Med. 2015; 8:14595–603. 26628942 PMC4658831

[21] Gui CP, Wei JH, Chen YH, Fu LM, Tang YM, Cao JZ, Chen W, Luo JH. A new thinking: extended application of genomic selection to screen multiomics data for development of novel hypoxia-immune biomarkers and target therapy of clear cell renal cell carcinoma. Brief Bioinform. 2021; 22:bbab173. 10.1093/bib/bbab173 34237133

[22] Rooney MS, Shukla SA, Wu CJ, Getz G, Hacohen N. Molecular and genetic properties of tumors associated with local immune cytolytic activity. Cell. 2015; 160:48–61. 10.1016/j.cell.2014.12.033 25594174 PMC4856474

[23] Şenbabaoğlu Y, Gejman RS, Winer AG, Liu M, Van Allen EM, de Velasco G, Miao D, Ostrovnaya I, Drill E, Luna A, Weinhold N, Lee W, Manley BJ, et al. Tumor immune microenvironment characterization in clear cell renal cell carcinoma identifies prognostic and immunotherapeutically relevant messenger RNA signatures. Genome Biol. 2016; 17:231. 10.1186/s13059-016-1092-z 27855702 PMC5114739

[24] Bui TO, Dao VT, Nguyen VT, Feugeas JP, Pamoukdjian F, Bousquet G. Genomics of Clear-cell Renal Cell Carcinoma: A Systematic Review and Meta-analysis. Eur Urol. 2022; 81:349–61. 10.1016/j.eururo.2021.12.010 34991918

[25] Ruffo E, Wu RC, Bruno TC, Workman CJ, Vignali DAA. Lymphocyte-activation gene 3 (LAG3): The next immune checkpoint receptor. Semin Immunol. 2019; 42:101305. 10.1016/j.smim.2019.101305 31604537 PMC6920665

[26] Chen B, Chen W, Jin J, Wang X, Cao Y, He Y. Data Mining of Prognostic Microenvironment-Related Genes in Clear Cell Renal Cell Carcinoma: A Study with TCGA Database. Dis Markers. 2019; 2019:8901649. 10.1155/2019/8901649 31781309 PMC6875323

[27] Saleh RR, Peinado P, Fuentes-Antrás J, Pérez-Segura P, Pandiella A, Amir E, Ocaña A. Prognostic Value of Lymphocyte-Activation Gene 3 (LAG3) in Cancer: A Meta-Analysis. Front Oncol. 2019; 9:1040. 10.3389/fonc.2019.01040 31681578 PMC6803551

[28] Zelba H, Bedke J, Hennenlotter J, Mostböck S, Zettl M, Zichner T, Chandran A, Stenzl A, Rammensee HG, Gouttefangeas C. PD-1 and LAG-3 Dominate Checkpoint Receptor-Mediated T-cell Inhibition in Renal Cell Carcinoma. Cancer Immunol Res. 2019; 7:1891–9. 10.1158/2326-6066.CIR-19-0146 31484656

[29] Sauer N, Szlasa W, Jonderko L, Oślizło M, Kunachowicz D, Kulbacka J, Karłowicz-Bodalska K. LAG-3 as a Potent Target for Novel Anticancer Therapies of a Wide Range of Tumors. Int J Mol Sci. 2022; 23:9958. 10.3390/ijms23179958 36077354 PMC9456311

[30] Sun D, Wang J, Han Y, Dong X, Ge J, Zheng R, Shi X, Wang B, Li Z, Ren P, Sun L, Yan Y, Zhang P, et al. TISCH: a comprehensive web resource enabling interactive single-cell transcriptome visualization of tumor microenvironment. Nucleic Acids Res. 2021; 49:D1420–30. 10.1093/nar/gkaa1020 33179754 PMC7778907

[31] Ru B, Wong CN, Tong Y, Zhong JY, Zhong SSW, Wu WC, Chu KC, Wong CY, Lau CY, Chen I, Chan NW, Zhang J. TISIDB: an integrated repository portal for tumor-immune system interactions. Bioinformatics. 2019; 35:4200–202. 10.1093/bioinformatics/btz210 30903160

[32] Li T, Fan J, Wang B, Traugh N, Chen Q, Liu JS, Li B, Liu XS. TIMER: A Web Server for Comprehensive Analysis of Tumor-Infiltrating Immune Cells. Cancer Res. 2017; 77:e108–10. 10.1158/0008-5472.CAN-17-0307 29092952 PMC6042652

[33] Yoshihara K, Shahmoradgoli M, Martínez E, Vegesna R, Kim H, Torres-Garcia W, Treviño V, Shen H, Laird PW, Levine DA, Carter SL, Getz G, Stemke-Hale K, et al. Inferring tumour purity and stromal and immune cell admixture from expression data. Nat Commun. 2013; 4:2612. 10.1038/ncomms3612 24113773 PMC3826632

[34] Xu L, Deng C, Pang B, Zhang X, Liu W, Liao G, Yuan H, Cheng P, Li F, Long Z, Yan M, Zhao T, Xiao Y, Li X. TIP: A Web Server for Resolving Tumor Immunophenotype Profiling. Cancer Res. 2018; 78:6575–80. 10.1158/0008-5472.CAN-18-0689 30154154

[35] Franceschini A, Szklarczyk D, Frankild S, Kuhn M, Simonovic M, Roth A, Lin J, Minguez P, Bork P, von Mering C, Jensen LJ. STRING v9.1: protein-protein interaction networks, with increased coverage and integration. Nucleic Acids Res. 2013; 41:D808–15. 10.1093/nar/gks1094 23203871 PMC3531103

[36] Warde-Farley D, Donaldson SL, Comes O, Zuberi K, Badrawi R, Chao P, Franz M, Grouios C, Kazi F, Lopes CT, Maitland A, Mostafavi S, Montojo J, et al. The GeneMANIA prediction server: biological network integration for gene prioritization and predicting gene function. Nucleic Acids Res. 2010; 38:W214–20. 10.1093/nar/gkq537 20576703 PMC2896186

[37] Stark C, Breitkreutz BJ, Reguly T, Boucher L, Breitkreutz A, Tyers M. BioGRID: a general repository for interaction datasets. Nucleic Acids Res. 2006; 34:D535–9. 10.1093/nar/gkj109 16381927 PMC1347471

[38] Lánczky A, Győrffy B. Web-Based Survival Analysis Tool Tailored for Medical Research (KMplot): Development and Implementation. J Med Internet Res. 2021; 23:e27633. 10.2196/27633 34309564 PMC8367126

