# Supplementary material for: Integrative analysis of LAG3 immune signature and identification of a LAG3-related genes prognostic signature in kidney renal clear cell carcinoma
Source: Aging (Albany NY). 2024 Jan 25;16(3):2161–80. doi: 10.18632/aging.205476 (PMC10911349; doi:10.18632/aging.205476)
Supplement: Supplementary Figures [file aging-16-205476-s001.pdf]

SUPPLEMENTARY FIGURES

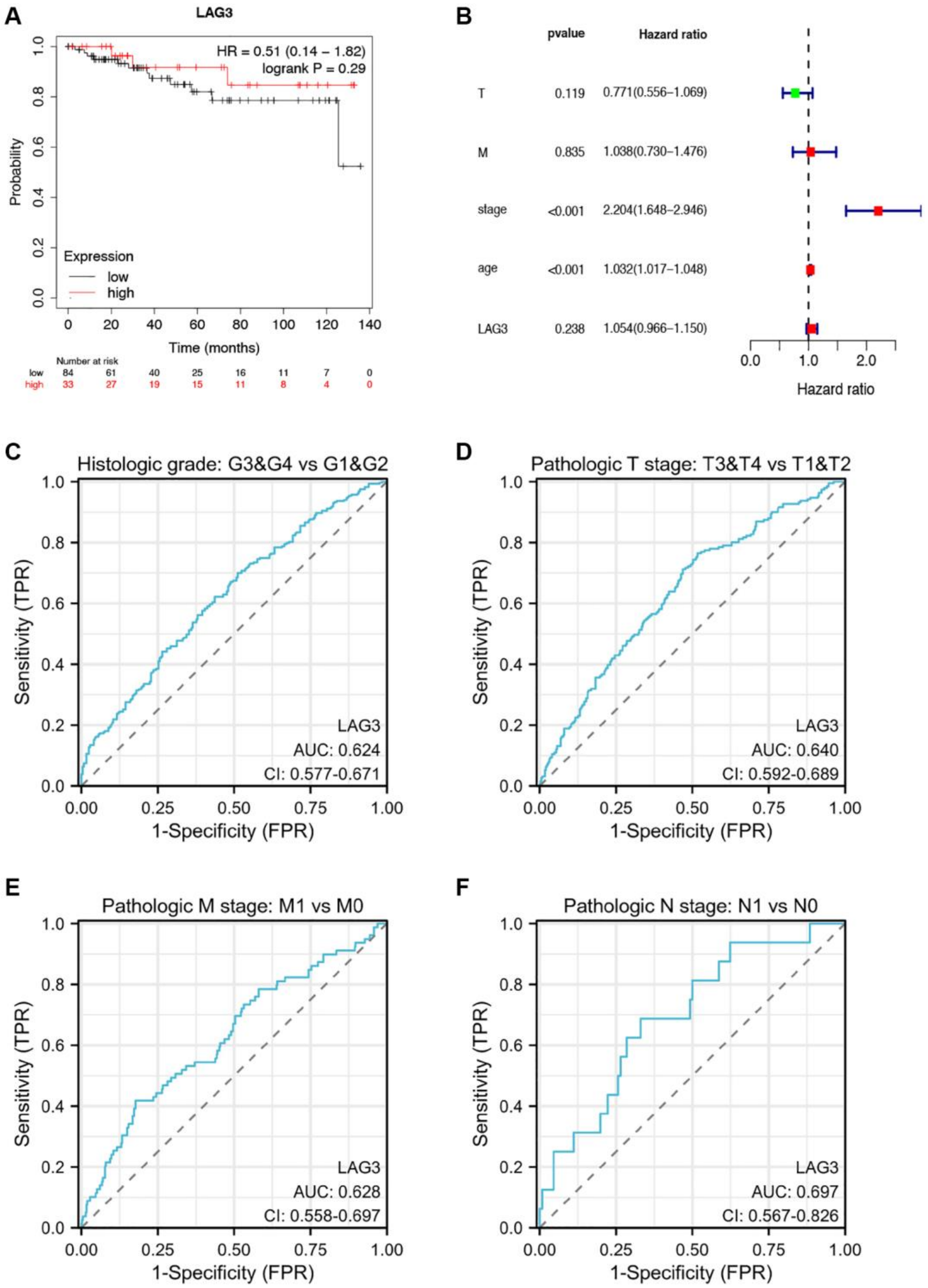

Supplementary Figure 1. The prognostic and diagnostic value of LAG3. (**A**) Kaplan–Meier curves of OS between high and low LAG3 expression groups. (**B**) Multivariate Cox regression analysis of LAG3. ROC curve to evaluate the diagnostic value of LAG3 expression in distinguishing different clinical and pathological features of KIRC, including pathologic stages (**C**), histologic grades (**D**), pathologic M stage (**E**), and pathologic N stage (**F**).

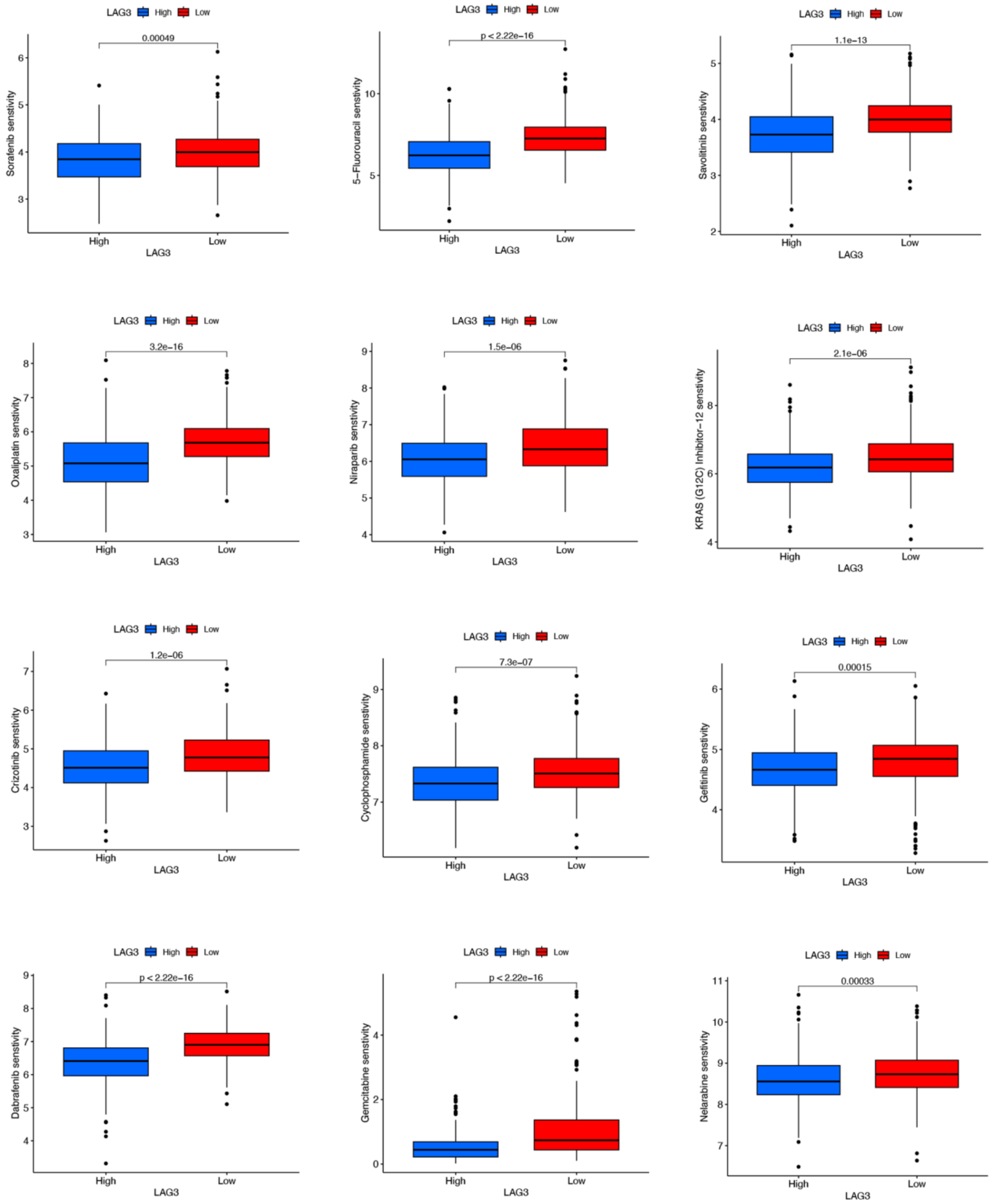

Supplementary Figure 2. Box plot of drug sensitivity difference between the high- and low-LAG3 expression groups.

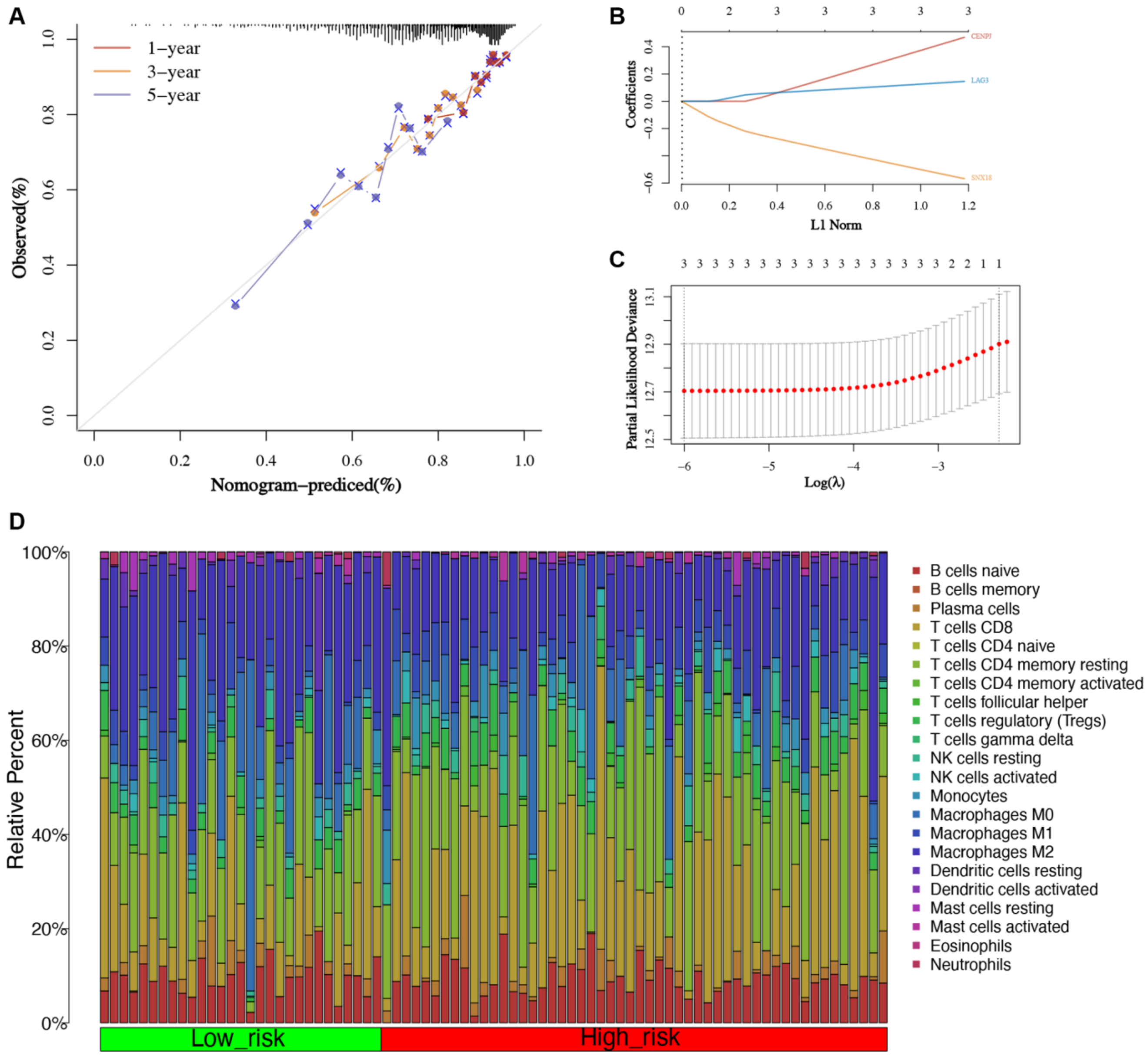

Supplementary Figure 3. (**A**) Nomogram calibration curve for the OS. LASSO Cox model coefficient (**B**) and partial likelihood deviance (**C**). (**D**) The bar graph illustrates the composition of immune cell proportions in the high and low-risk groups. ($^{*}P < 0.05$; $^{**}P < 0.01$; $^{***}P <$ 0.001).
