# Supplementary material for: Integrative analysis of LAG3 immune signature and identification of a LAG3-related genes prognostic signature in kidney renal clear cell carcinoma
Source: Aging (Albany NY). 2024 Jan 25;16(3):2161–80. doi: 10.18632/aging.205476 (PMC10911349; doi:10.18632/aging.205476)
Supplement: Supplementary Table 1 [file aging-16-205476-s002.pdf]

SUPPLEMENTARY TABLE

Supplementary Table 1. The 65 genes directly interacting with LAG3.

Gene	Attribute	Gene	Attribute	Gene	Attribute	Gene	Attribute
CD4	STRING	FGL1	GeneMINA	AGRV1	HitPredict	CHRNA5	BioGRID
HAVCR2	STRING	CENPJ	GeneMINA	CELR2	HitPredict	CELSR1	BioGRID
TIGIT	STRING	GZMK	GeneMINA	CELR1	HitPredict	PCDH1	BioGRID
LGALS3	STRING	WEIKKYZ	GeneMINA	DPA1	HitPredict	HLA-DPA1	BioGRID
CTLA4	STRING	IRF4	GeneMINA	FAT1	HitPredict	PCDH7	BioGRID
SNCA	STRING	POU2F2	GeneMINA	FREM2	HitPredict	LRP5	BioGRID
LAG3	STRING	IL2RB	GeneMINA	PCDH7	HitPredict	ITIH2	BioGRID
CD274	STRING	CCR5	GeneMINA	CENPJ	HitPredict	COL12A1	BioGRID
FGL1	STRING	L2RA	GeneMINA	ITIH2	HitPredict	FBXO27	BioGRID
PDCD1	STRING	LAG3	GeneMINA	SNX18	HitPredict	FBXO11	BioGRID
CLEC4G	STRING	LL15RA	GeneMINA	FBX2	HitPredict	SNX18	BioGRID
		FLT3LG	GeneMINA	C1QRF	HitPredict	CELSR2	BioGRID
		SYTL1	GeneMINA	PCD20	HitPredict	LAG3	BioGRID
		PDLM2	GeneMINA	FBX11	HitPredict	CBWD3	BioGRID
						SKP1	BioGRID
						PCDH20	BioGRID
						FREM2	BioGRID
						FRAS1	BioGRID
						C1QL1	BioGRID
						GPR98	BioGRID
						PTPRK	BioGRID
						FAT1	BioGRID
						CBWD1	BioGRID
						FBXO2	BioGRID
						CSPG4	BioGRID
						MANBA	BioGRID
